# Colloidal lithography for fabricating patterned polymer-brush microstructures

**DOI:** 10.3762/bjnano.3.46

**Published:** 2012-05-15

**Authors:** Tao Chen, Debby P Chang, Rainer Jordan, Stefan Zauscher

**Affiliations:** 1 Department of Chemie, Technische Universität Dresden, 01069 Dresden, Germany; 2 Center for Biologically Inspired Materials and Materials Systems, and Department of Mechanical Engineering and Materials Science, Duke University, Durham, NC, 27708, USA; 3 Department of Physical Chemistry, Lund University, SE-221 00 Lund, Sweden

**Keywords:** atom-transfer radical polymerization, colloidal lithography, patterning, self-assembled microsphere monolayer

## Abstract

We exploit a series of robust, but simple and convenient colloidal lithography (CL) approaches, using a microsphere array as a mask or as a guiding template, and combine this with surface-initiated atom-transfer radical polymerization (SI-ATRP) to fabricate patterned polymer-brush microstructures. The advantages of the CL technique over other lithographic approaches for the fabrication of patterned polymer brushes are (i) that it can be carried out with commercially available colloidal particles at a relatively low cost, (ii) that no complex equipment is required to create the patterned templates with micro- and nanoscale features, and (iii) that polymer brush features are controlled simply by changing the size or chemical functionality of the microspheres or the substrate.

## Introduction

It is well known that monodisperse colloidal microspheres easily self-assemble into hexagonally close-packed arrays on surfaces as a result of capillary forces arising from the evaporation of solvents [1–4]. Such periodic arrays of microspheres were used already in the early 1980s by Fischer and co-workers as shadow masks in colloid lithography (CL) for the deposition of platinum nanomaterials [5]. Since then, CL has become a simple, versatile, and cost-effective fabrication technique for a large number of researchers in the field of micro/nanofabrication [2–4,6]. A variety of lithographic methods have since been developed, in which colloid microsphere arrays are used as masks for depositing nanomaterials and as scaffolds for templating 2-D or 3-D functional patterns [2–5,7–9]. When a 2-D colloidal crystal array is used as a shadow mask in metallic vapor deposition, the metal deposited by sputtering can reach the substrate only through the interstices between the spheres, and the shape of the deposits on the substrate is thus determined by the projected area of the interstices on the substrate [2,4]. Micro- and nanospheres can also be used to guide the transport of molecules so that the molecular deposition forms a ring-shaped pattern around the contact point (footprint) of the microsphere with the substrate [9]. For a self-assembled microsphere monolayer (SMM) on a substrate, the footprint between the microsphere and substrate produces a barrier array, which can be used as a template for lithography [6,10–11]. CL thus provides a straightforward way to adjust the feature size at the microscale and, by using sufficiently small spheres, the nanoscale, by changing the sphere diameter of the colloid mask. Spherical particles are commercially available with a wide range of sizes and types, or can be synthesized, e.g., by emulsion polymerization for polymer latex spheres or by controlled precipitation for inorganic oxides [12]. Patterned polymer brushes [13] are of increasing importance especially for array-based platforms because of their ability to modify surface properties and their potential applications in surface-based technologies, such as protein-resistant coatings, switchable sensors, substrates for cell-growth control, and for the separation of biological molecules [14–16]. They can be grown by surface-initiated polymerization from surface-confined initiator templates, as fabricated by various lithographic approaches. Although a range of strategies for polymer brush patterning, including photolithography [17], electron-beam lithography [18], electron-beam chemical lithography [19], microcontact printing (µCP) [20], scanning-probe lithography [21] and capillary-force lithography [22], have been exploited over the years, there is still considerable interest in the exploitation of new, simple patterning strategies that do not entail instrumental complexity. As an inexpensive alternative to conventional lithography, CL provides new possibilities to create patterned polymer brushes. So far only one of the CL strategies, using the SMM footprint as the mask, has been demonstrated for fabricating patterned pillar [23] or cavity [11,24] polymer brushes, and we recently reported how SMM could be used as µCP stamps to fabricate cone-shaped polymer brushes [25].

In this letter we report how we exploit a range of robust and simple patterning strategies offered by colloidal lithography, and combine them with surface-initiated atom-transfer radical polymerization (SI-ATRP) for patterning polymer-brush microstructures. The use of CL for patterning polymer brushes has significant advantages over the lithographic approaches mentioned above, in that it employs commercially available, relatively low cost nano- and microspheres, that it does not require complex equipment to create micro- and nanopatterned templates, and in that it allows control over polymer-brush geometry by simple changing of the diameter or chemical functionality of the nano- or microspheres. A recent paper [6] showed that colloidal particles on the order of 100 nm can be used to pattern silane features with nanometer dimensions. Due to the similarity of this and our patterning approach, we do not foresee a problem in scaling down our approach shown here, to fabricate polymeric nanostructures with lateral feature dimensions on the order of 100 nm.

## Results and Discussion

**Figure 1:**
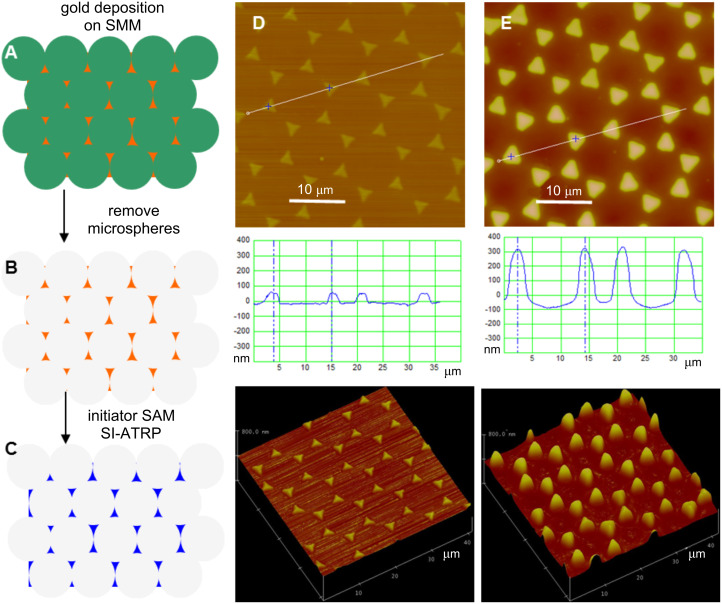
Schematic illustration and AFM images showing the use of CL in the fabrication of patterned polymer-brush microstructures. (A) SMM on a silica wafer serves as a template for gold deposition. (B) Removal of the microspheres by sonication. (C) Functionalization of the Au pattern with a thiol initiator SAM and subsequent amplification into polymer brushes. (D, E) Contact-mode AFM height images (40 μm × 40 μm, imaged at room temperature in air) of the patterned gold islands before and after PNIPAAM brush growth, and the corresponding height profiles and 3-D images.

Hexagonally packed arrays of self-assembled colloidal micro- and nanospheres on surfaces have been used as masks to guide deposition or etching through the interstices between the colloidal microspheres [5–6,9]. For example, arrays of triangularly shaped metal islands can be obtained by sputter deposition of the metal [2,4]. When gold is chosen as the metal, the ensuing pattern can be easily functionalized chemically with a self-assembled monolayer (SAM) of a thiol initiator, which can be subsequently amplified into polymer brushes. Figure 1 shows this strategy for the patterning of colloidal microspheres for the fabrication of polymer-brush microstructures. We first assembled a SMM of polystyrene latex (diameter ≈ 10 µm) on a silica substrate by gravity-induced sedimentation combined with solvent evaporation [26], and subsequently we deposited gold into the interstices between the microspheres (Figure 1A). After the microsphere mask was removed by sonication, an array of hexagonally arranged triangular gold islands remained (Figure 1B) on which we formed a SAM of thiol initiator (BrC(CH_3_)_2_COO(CH_2_)_11_SH) [27]. We then synthesized poly(*N*-isopropylacrylamide) (PNIPAAM) brush microstructures on the islands by SI-ATRP of NIPAAM (Figure 1C). An AFM image of the patterned gold islands reveals a feature height of about 65 nm (Figure 1D). The feature size of a triangular island (≈2.3 µm) is about one quarter of the sphere diameter, and the distance between nearest-neighbor islands (≈5.3 µm) is around half of the sphere diameter, in accordance with a previous report by Haynes et al. [7]. The resulting PNIPAAM brush height was about 350 nm, and due to polymerization also occurring at the sides of the triangles, the footprint size increased to about 2.9 µm (Figure 1E) while the distance between nearest-neighbor islands remained about 5.3 μm. The feature size of the polymer brushes can be altered by changing (i) the size of the microspheres, (ii) the assembly of the spheres on the substrate surface, or by (iii) varying the conditions of the gold vapor deposition, to yield a range of microstructures [28].

**Figure 2:**
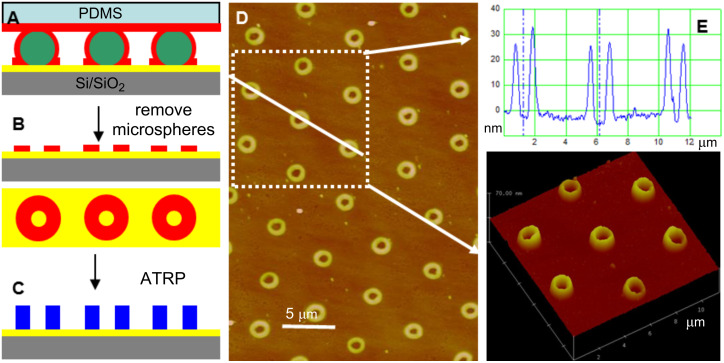
Schematic illustration and AFM images showing the use of ESL for the fabrication of ring-shaped polymer-brush microstructures. (A) Arrayed SMM direct the transport of alkanethiol initiator from a planar PDMS stamp onto the gold surface (printing was carried out by gently pressing the PDMS stamp onto the SMM template for 30 s). (B) Ring-shaped SAM formed after removal of the microspheres. (C) Subsequent amplification into hollow polymer-brush cylinders. (D,E) Contact-mode AFM height images of the patterned PNIPAAM brush microstructures imaged at room temperature in air, and the corresponding height profiles and 3-D image.

Colloidal microspheres have an inherently curved surface that can serve as a template for spreading alkanethiol molecules along the surface of the microspheres onto the gold substrate surface, creating a ring-shaped SAM feature around the footprint of the sphere–surface contact area. This so-called edge-spreading lithography (ESL) employing colloid microspheres as templates has been previously used to fabricate ring-shaped metal patterns [9]. Here we replaced the octadecanethiol (ODT) molecules with thiol initiator (BrC(CH_3_)_2_COO(CH_2_)_11_SH), and amplified the annular thiol initiator monolayer into ring-shaped polymer brushes (Figure 2). In this patterning approach we used a SMM (sphere diameter ≈ 5 μm) to direct the transport of an alkanethiol initiator from an initiator-inked planar poly(dimethyl siloxane) (PDMS) stamp onto the gold surface (Figure 2A). Upon reaching the metal substrate, the thiol initiator molecules self-assemble into a patterned monolayer, which is confined by the footprint of each microsphere and the extent of lateral spreading of the thiols on the gold substrate (Figure 2B). Amplification of the ring-shaped initiator SAMs results in patterned, hollow cylindrical polymer brushes (Figure 2C–E). The inner diameter of the polymer-brush cylinders is about 900 nm. This diameter reflects the underlying ring-shaped initiator pattern and is on the order of 18% of the microsphere diameter, in close agreement with a previous report [9]. The outer diameter of the hollow polymer-brush cylinders is about 1.5 µm, and is largely determined by the contact time of the PDMS stamp on the microsphere template, which implies that the diffusion of the thiol initiator along the surface of each microsphere depends on the contact time with the PDMS stamp [9]. Furthermore, polymer brush microstructures may be varied by changing the concentration of the thiol initiator, or by adding inert thiol molecules [29], which affects the thiol initiator distribution and diffusion on the gold surface.

**Figure 3:**
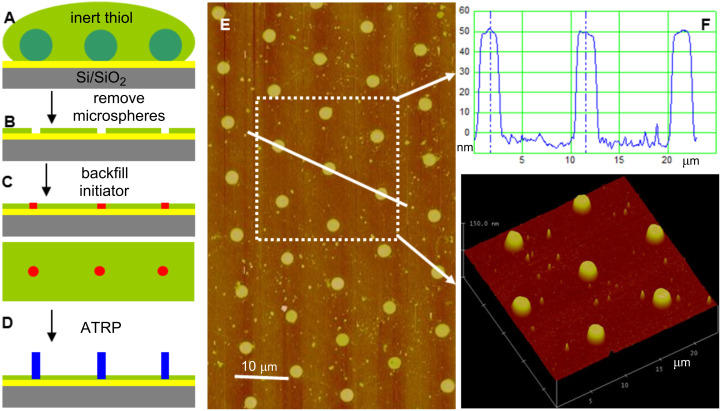
Schematic illustration and AFM images showing use of colloidal microsphere lithography for patterning polymer-brush pillars. (A) A SMM, assembled on a gold substrate, serves as a mask for the inert thiol SAM pattern. (B) After ink transfer and drying in nitrogen, the polystyrene microsphere mask was removed, leaving an inert thiol SAM pattern. (C) The substrate was then backfilled with thiol initiator. (D) Subsequent pattern amplification into polymer-brush microstructure by SI-ATRP of NIPAAM. (E, F) Contact-mode AFM height images of patterned PNIPAAM-brush microstructure imaged at room temperature in air and the corresponding height profiles and 3-D image.

Our results show that microspheres can be used to guide the spreading of a thiol initiator to form ring-shaped thiol patterns around the footprint of microspheres on the surface. While initiator-inked stamps only provide a limited thiol reservoir, the microsphere footprint could also be used as a mask for fabricating polymer-brush pillars, by inking the microsphere array with a large amount of thiol. Such an approach was first reported by Taylor and co-workers [10], who described a simple CL technique to fabricate substrates with hexagonally patterned dots of protein surrounded by a protein-repellant layer of poly(ethylene glycol) (PEG). In that work, a self-assembled monolayer of latex spheres served as a lithographic mask to selectively graft a thin layer of PEG around the footprint of the microspheres. After removal of the spheres, a periodic pattern of holes in the protein-repellant PEG layer was exposed, and proteins could be selectively adsorbed onto the underlying surface in these holes. In a similar approach we used inert thiol to cover a SMM of polystyrene microspheres (diameter ≈ 10 μm) (Figure 3A) to form an inert thiol SAM everywhere except in the footprint of each microsphere (Figure 3B), and then backfill with a thiol initiator (Figure 3C). Amplification of this pattern, after removal of the SMM, resulted in a periodic pattern of polymer-brush pillars (about 50 nm high and about 1.5 μm in diameter, Figure 3D–F). The diameters of the polymer cylinders were on the order of 15% of the microsphere diameter, in agreement with our result described above (ca. 18%).

**Figure 4:**
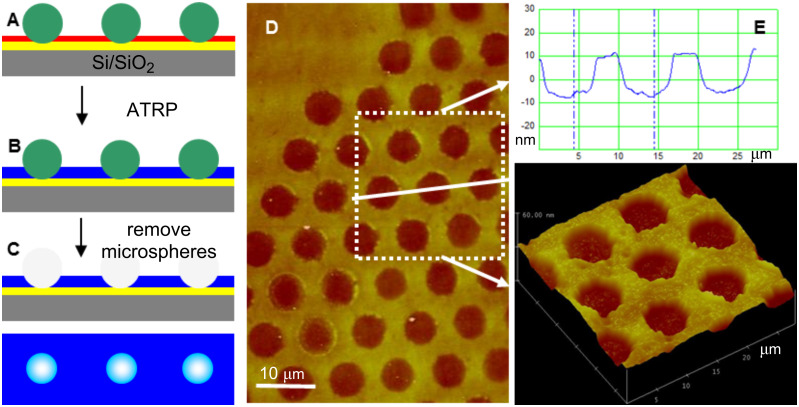
Schematic illustration and AFM images showing the use of colloidal microsphere lithography for patterning hole-like polymer-brush microstructures. (A) SMM on thiol initiator SAM-coated gold substrate. (B) Subsequent pattern amplification into polymer-brush microstructure by SI-ATRP of NIPAAM. (C) Removal of the polystyrene microsphere mask leaves a hole-patterned brush thin film. (D, E) Contact-mode AFM height images of hole-patterned PNIPAAM-brush thin film imaged at RT in air and the corresponding height profiles and 3-D image.

Another type of polymer-brush microstructure can be designed by inking the microsphere arrays by thiol initiator first, to form an initiator SAM around the microspheres. This should result in a polymer-brush layer with a patterned hole-like microstructure after removal of the microspheres and subsequent amplification [11]. Xu et al. developed a method to pattern a surface with polymer brushes during a polymerization process in a microchannel, formed between PDMS stamps and initiator-modified substrates [30]. This so-called microchannel-confined surface-initiated polymerization technique showed that there is no polymer brush growth in the contact area of the PDMS stamp with an initiator-functionalized SAM-coated silicon wafer. This inspired us to form a SMM on thiol-initiator-coated gold substrates as a template for fabricating hole-patterned polymer brushes. We first assembled a mask of polystyrene latex particles (SMM) on a gold substrate previously covered with a SAM of thiol initiator (Figure 4A), and then amplified the exposed initiator by SI-ATRP of NIPAAM (Figure 4B). After removing the SMM, a polymer-brush thin film with a hole pattern was obtained (Figure 4C,D). The patterned polymer brush layer has a height of about 16 nm and a hole diameter of about 6 μm.

## Conclusion

In summary, we have demonstrated how colloidal lithography can provide a simple approach with various strategies to fabricate a range of patterned polymer-brush microstructures. Our approaches rely on the spontaneous formation of well-ordered, colloidal microsphere arrays that provide lithographic masks, templates, and footprint-restricted geometries for creating patterns of initiator SAMs that can be used for subsequent amplification into polymer-brush patterns. Compared with other lithographic techniques to fabricate patterned polymer brushes, CL has the advantage of (i) not requiring any special instrumentation and (ii) changing feature size simply by changing the microsphere diameters used in the colloid masks, or changing the colloid deposition parameters. Patterned polymer brushes are of increasing importance for array-based platforms and applications in surface-based technologies, such as protein-resistant coatings, switchable sensors, substrates for cell-growth control, and for separation of biological molecules. We note that for convenience and proof-of-concept of our approach, we used PS microspheres to fabricate patterned polymer brushes with lateral feature dimensions on the micrometer and submicrometer length scales. A recent paper [6] shows, that colloidal particles on the order of 100 nm can be used to pattern silane features with nanometer dimensions. Due to the similarity of this and our approach, we do not foresee a problem in scaling our approach down to fabricate polymer nanostructures with lateral feature dimensions on the order of 100 nm.

## Experimental

**Materials:** *N*-isopropylacrylamide (NIPAAM) (99%), copper(I) bromide (CuBr, 99.9%), methanol (MeOH, 99.9%) and ethanol were obtained from Sigma-Aldrich (Milwaukee, WI). Milli-Q (Millipore, Billerica, MA) water (18 MΩ·cm) and methanol were used as polymerization solvents. *N*,*N′*,*N′*,*N″*,*N″*-Pentamethyldiethylenetriamine (PMDETA) was used as received from Acros Organics (Hampton, NH). The thiol initiator (BrC(CH_3_)_2_COO(CH_2_)_11_SH) was synthesized as reported [27]. Polystyrene microspheres (5 µm and 10 µm) were donated by Dr. R. M. Erb at Duke University, who purchased them from Duke Scientific Corporation (Palo Alto, CA). To immobilize the initiators for surface-initiated polymerization, gold substrates with an average grain diameter of 60 nm were prepared by thermal evaporation under a vacuum of 4 × 10^−7^ Torr. For this purpose an adhesion layer of chromium (50 Å) followed by a layer of gold (600 Å) was evaporated onto silicon wafers. Before deposition, silicon wafers were cleaned in a mixture of H_2_O_2_/H_2_SO_4_ (1:3, v/v) at 80 °C (“piranha solution”) for 10 min and washed thoroughly with Milli-Q-grade water. (Caution: Piranha solution reacts violently with organic matter!)

**SMM on silica substrate:** After the polystyrene microspheres were transferred from aqueous suspension (0.5 mL) into ethanol (1.0 mL) with subsequent shaking, they were first centrifuged and then the mixed solvent was removed. The residual was then redispersed in ethanol (0.5 mL) for subsequent pipetting onto a slightly tilted silica wafer. Upon drying at room temperature the microspheres self-assembled to form regions of hexagonally close-packed monolayers by gravity-induced sedimentation combined with solvent evaporation [1,26].

**Deposition of gold on SMM-coated silica substrate:** The procedure of gold coating on SMM covered silica wafers was similar to that used for the gold coating of the silicon wafers. A subsequent sonication was used to remove the polystyrene microspheres and leave an array of triangular gold dots.

**ESL from a flat PDMS stamp using SMM as a mask:** Inking was done by covering a stamp with a solution of 2 mM thiol-initiator/ethanol solution for 1 min, and drying the stamp in a stream of nitrogen. Printing was carried out gently by hand onto SMM-constructed gold-coated silica wafer for 30 s. Microspheres were then removed prior to polymerization by sonication in a deionized water bath for about 2 min.

**Thiol-initiator monolayer preparation:** Gold-coated silica wafer was put into an ethanol solution of thiol initiator (ca. 2 mM) overnight and then removed and dried with nitrogen.

**SMM on initiator-monolayer-coated gold substrate:** After the polystyrene microspheres were transferred from aqueous suspension (0.5 mL) into ethanol (1.0 mL) with a subsequent shake, they were first centrifuged and then the mixed solvent was removed. The residual was then redispersed in ethanol (0.5 mL) for subsequent pipetting onto a slightly tilted initiator-coated gold substrate wafer. Upon drying at room temperature the microspheres self-assembled to form regions of hexagonally close-packed monolayers by gravity-induced sedimentation combined with solvent evaporation.

**SI-ATRP:** The polymer brushes were prepared according to our previous procedures with some slight modifications [31]. Briefly, the polymerization solution was prepared by adding a solution of NIPAAM monomer to an organometallic catalyst. The organometallic catalyst was formed in a nitrogen atmosphere by adding CuBr (1.8 mg, 0.013 mmol) and PMDETA (14 µL, 0.064 mmol) in a 1:5 molar ratio to 1.0 mL of MeOH as solvent. The mixture was then sonicated for 1–2 min to facilitate the formation of the CuBr/PMDETA complex. Next, 1.5 g (17 mmol) of NIPAAM monomer dissolved in 5 mL of water was filtered into the catalyst-complex solution through a 0.45 μm Millipore Millex filter. The polymerization solution was then transferred into flasks containing the sample substrates with the immobilized patterned initiator. The flasks were sealed with rubber septa and kept at room temperature under nitrogen. After the desired reaction time, substrates were removed from the polymerization solution, exhaustively rinsed with deionized water to remove all traces of the polymerization solution, and dried in a stream of nitrogen.

**Characterization:** The patterned polymer-brush microstructure samples were rinsed with Milli-Q-grade water, dried under a stream of nitrogen, and mounted on steel sample disks prior to AFM measurements. AFM topographic images were collected in contact mode by using V-shaped silicon nitride cantilevers (Nanoprobe, Veeco, spring constant 0.12 N/m; tip radius 20–60 nm) using a MultiMode atomic force microscope (Digital Instruments, Santa Barbara, CA). The AFM topographic images performed in air, were obtained under low applied normal forces (<1 nN) to minimize compression and lateral damage of the polymer brushes. The relatively large lateral size of the polymer-brush features did not necessitate image deconvolution to account for tip-induced broadening of the feature dimensions [32].
